# Automatic analysis of speech representations to assess psychological distress

**DOI:** 10.3389/fdgth.2026.1886893

**Published:** 2026-08-12

**Authors:** Sara Fernández-Velasco, Jose Moreno-Mesa, Daniel Escobar-Grisales, Diego M. Lopez, Juan Rafael Orozco-Arroyave

**Affiliations:** 1 Telematics Department, Universidad del Cauca, Popayán, Colombia; 2 GITA Lab, Universidad de Antioquia, Medellín, Colombia

**Keywords:** DAIC-WOZ, depression, digital mental health, participant-level validation, psychological distress, post-traumatic stress disorder, speech analysis, vocal biomarkers

## Abstract

**Background:**

Current mental health diagnostic methods are limited by subjective clinical interpretation. Automatic speech analysis is a promising technology for objective assessment.

**Objective:**

To evaluate and compare different speech-based representations (acoustic, phonetic, and time-frequency) and deep learning-based embeddings for discriminating symptoms associated with psychological distress.

**Methods:**

A secondary analysis of the Distress Analysis Interview Corpus (DAIC-WOZ) was conducted using recordings from 125 participants (3,069 responses). Speech representations included phonation, articulation, and prosody features extracted with DisVoice; phonetic features extracted with Phonet; time-frequency representations derived from Mexican hat wavelets; and deep embeddings extracted with the multilingual Wav2Vec 2.0 model XLSR-53. Two classification strategies were addressed at the response and participant levels using a Fully Connected Neural Network (FCNN) and a Support Vector Machine (SVM), respectively.

**Results:**

Prosody at the participant level achieved the highest mean performance (F1-score 0.67 ± 0.07; accuracy 0.64 ± 0.10; AUC 0.65 ± 0.12), followed by participant-level phonation (F1-score 0.59 ± 0.16; accuracy 0.61 ± 0.14; AUC 0.65 ± 0.16). Conversely, participant-level aggregation of deep embeddings yielded lower performance (F1-score 0.48 ± 0.19; accuracy 0.55 ± 0.13; AUC 0.52 ± 0.15), failing to surpass traditional features. Response-level performance remained close to chance. Phonet and wavelet representations did not improve performance over prosody or phonation.

**Conclusion:**

Participant-level analysis provided more robust and consistent discriminative patterns than response-level approaches. Prosody and phonation achieved the best performance across speech representations, while phonetic, time-frequency, and deep speech representations did not outperform the best acoustic baseline. These findings suggest that, within the evaluated experimental setting, the aggregation strategy appears to have a stronger influence on performance than increasing representational complexity.

## 1 Introduction

According to the World Health Organization (WHO), mental disorders affect a substantial portion of the global population and contribute significantly to disability and reduced quality of life [1]. Conditions such as depression and post-traumatic stress disorder (PTSD) are commonly assessed through clinical interviews, self-report questionnaires, and behavioral observation. Although these approaches are widely used, they rely heavily on subjective clinical interpretation and are sensitive to patients’ willingness to disclose information. These limitations may delay diagnosis, negatively impacting patients’ quality of life, and increase the burden on healthcare systems [2]. In recent years, different physiological and behavioral signals have been explored as complementary sources of information for mental health assessment, including electroencephalography, facial expressions, heart-rate variability, and speech, with the aim of providing objective information to support the identification of these conditions.

Automatic speech analysis has emerged as a promising approach to support mental health assessment. Speech production is influenced by cognitive and emotional states, making it a relevant source of behavioral biomarkers. In this context, Scherer et al. [3] demonstrated that vocal behavior contains relevant information about the emotional state of the speaker. In particular, [4] showed that variations in pitch and speech rate may reflect depressive symptoms, while Cummins et al. [5] reported that changes in intonation, energy, and rhythm are useful for detecting PTSD. Similarly, [6] analyzed acoustic speech features, including phonation- and articulation-related characteristics, highlighting their importance in the automatic identification of psychiatric disorders. More recently, studies such [7] have explored more complex speech representations using deep learning approaches and time-frequency analysis, aiming to capture temporal and spectral patterns associated with mental health conditions.

Despite the growing interest in automatic speech analysis to support mental health assessment, the availability of clinically validated speech corpora for mental health assessment remains limited. Among the few open clinical corpora available is the Distress Analysis Interview Corpus (DAIC), introduced by Gratch et al. [8], which provides audio recordings, transcripts, physiological signals and clinical assessments obtained from semi-structured interviews. In this corpus, distress is understood as the presence of symptoms associated with depression, PTSD, or both. Studies using this dataset, such as Williamson et al. [9] and Al Hanai et al. [10], have explored acoustic representations and deep learning strategies for the detection of depressive symptoms. Additionally, multimodal approaches combining speech with text or other signals have reported promising results for mental health assessment. However, these studies mainly focused on specific modeling strategies or modalities, with limited attention to systematically comparing heterogeneous speech representations under unified response- and participant-level evaluation settings.

Another unresolved issue in the aforementioned studies is how speech responses should be aggregated and evaluated when clinical labels are defined at the participant level, as in the DAIC-WOZ dataset. This aspect is particularly relevant in the context of psychological distress, where depression and PTSD frequently co-occur and may share speech-related manifestations in trauma-exposed populations.

To address the above limitations, this study compares acoustic, phonetic, time-frequency, and deep learning-based speech representations for distinguishing healthy controls from participants with distress-related mental health conditions in the DAIC-WOZ dataset. Models were evaluated at both the response and participant levels using subject-stratified cross-validation to prevent participant overlap between training and test partitions. Rather than proposing a multimodal system, this work aims to clarify how the choice of speech representation and aggregation strategy influences discrimination performance in a realistic and heterogeneous interview setting.

## 2 Materials and methods

### 2.1 Dataset

This study is based on a secondary analysis of the DAIC-WOZ dataset [8], which consists of semi-structured clinical interviews aimed at assessing psychological distress. The dataset includes recordings of physiological signals, audio and video recordings of interactions between participants and a virtual interviewer, as well as corresponding transcripts and self-reported clinical assessments based on well-known clinical instruments including the Patient Health Questionnaire-8 (PHQ-8) for depression symptom severity and the PTSD Checklist-Civilian Version (PCL-C) for post-traumatic stress symptom severity. Since the aim of this study is on speech analysis exclusively, only participant speech responses were used. Speech segments with the interviewer’s speech were removed. Participants include U.S. military veterans and civilians from the general population. The DAIC-WOZ dataset is available upon request through the USC DCAPS repository.^1^

In this work, a subset of the dataset consisting of audio recordings with available and correctly aligned transcripts (n=186) was used, enabling the extraction of participant speech segments. The initial class distribution included 121 healthy controls and 65 participants with symptoms associated with depression, PTSD, or both. Following the terminology adopted in the original DAIC framework [8], these participants are hereafter referred to as the psychological distress group. To obtain a balanced participant-level sample, healthy controls were selected using random undersampling at the participant level from the 121 available healthy participants. A fixed random seed (random_state = 42) was used to ensure reproducibility of the sampling procedure. The selection considered the demographic variables available in the DAIC-WOZ dataset, namely age and gender. Although exact demographic matching was not feasible due to the broad age range represented in the corpus, the resulting subset maintained a demographic composition comparable to that of the psychological distress group. The resulting balanced sample consisted of 65 healthy controls and 65 participants with psychological distress (n=130), comprising 10,314 speech responses (5,321 from healthy controls and 4,993 from the psychological distress group). Subsequently, only speech chunks longer than 5 s were retained to ensure sufficient signal length for feature extraction, as several acoustic and phonetic descriptors employed in this study require speech segments of sufficient duration to generate stable and reliable representations. After applying this criterion, 3,607 healthy-control responses and 3,638 psychological-distress responses were excluded. The final dataset included 125 participants, including 65 healthy controls and 60 participants presenting psychological distress, and contributed a total of 3,069 responses (1,714 from healthy controls and 1,355 from the psychological distress group). The five excluded participants belonged to the psychological distress group and were excluded because no speech segments satisfying the minimum duration requirement remained available for analysis. Within the distress group, 7 participants exhibited depressive symptoms only, 17 PTSD symptoms only, and 36 both disorders. According to the PHQ-8 and PCL-C annotations available in the DAIC-WOZ corpus, the psychological distress group showed a mean PHQ-8 score of 13.38±4.67 and a mean PCL-C score of 54.08±11.89. In contrast, healthy controls showed mean PHQ-8 and PCL-C scores of 3.31±3.24 and 25.47±8.78, respectively. Demographic and dataset characteristics of the final sample are summarized in Table 1.

**Table 1:** Demographic and dataset characteristics of the final sample after filtering responses longer than 5 s.

Variable	Healthy controls	Psychological distress	Total
Participants, *n*	65	60	125
Female, *n* (%)	33 (50.8)	33 (55.0)	66 (52.8)
Male, *n* (%)	32 (49.2)	27 (45.0)	59 (47.2)
Responses, *n*	1,714	1,355	3,069
Age (years), mean ± SD	37.85±7.89	39.65±11.66	38.71±9.88
Responses per participant, mean ± SD	26.37±19.30	22.58±19.59	24.55±19.45
Response duration (s), mean ± SD	7.52±2.49	7.33±1.97	7.44±2.27

The target variable was formulated as a binary classification problem distinguishing healthy controls from participants with psychological distress. Following the labeling scheme provided in the DAIC-WOZ dataset, participants presenting symptoms associated with depression, PTSD, or both were grouped into a single distress-related category. This formulation was adopted because the primary objective of the study was to distinguish participants presenting psychological distress from healthy controls rather than differentiate specific psychiatric disorders. Moreover, grouping these conditions into a single category allowed addressing the heterogeneous distribution of the dataset and jointly modeling different manifestations of psychological distress, rather than treating them as highly imbalanced independent classes. Furthermore, this approach is consistent with the broader psychological distress perspective described in the original DAIC-WOZ corpus publication.

### 2.2 Preprocessing

The preprocessing of the audio signals was carried out to ensure homogeneous conditions for feature extraction. Each audio file corresponded to an individual response from a participant, and responses were organized using participant-level identifiers (e.g., 300_1, 300_2), where the first index denotes the participant. Audio files were converted to mono (single-channel), resampled to 16 kHz (the standard input rate required for the pre-trained XLSR-53 architecture), and peak-normalized by adjusting the gain to 0 dBFS. All files were stored in WAV format. Responses shorter than 5 s were excluded, as described in Section 2.1. All processing was implemented in Python (version 3.13.3) using standard audio processing libraries on a Linux-based environment.

### 2.3 Feature extraction

Acoustic, phonetic, time-frequency, and deep learning-based speech representations were extracted for each audio recording. These features are grouped according to different speech production characteristics and are intended to model different speech patterns, including those associated to abnormal speech production due to paralinguistic phenomena.

#### 2.3.1 Acoustic and phonetic features

Acoustic and phonetic features were extracted using the DisVoice and Phonet toolkits introduced by GITA Lab in [11, 12], which were specifically designed for automatic pathological speech analysis. In DisVoice, features are organized into three main dimensions: prosody, phonation, and articulation.

Prosodic features aim to model timing, intonation, and loudness patterns during speech production. In this dimension, 13 descriptors are computed in the voiced segments.

Phonation features are designed to characterize abnormal vocal fold vibration patterns. The feature set is composed of seven descriptors extracted from frames of 40 ms with a time-shift of 20 ms.

Articulation features aim to model the ability of the speaker to coordinate and move the muscles involved in the vocal tract. This representations is composed by 58 descriptor computed for frames of 40 ms of onset segments.

In addition to these acoustic representations, phonological features were extracted using Phonet [12]. This toolkit estimates phonological posteriors using bidirectional Gated Recurrent Units (GRUs). Phonemes are grouped into 18 phonological classes according to their manner and mode of articulation. Therefore, the posterior probabilities can be interpreted as a measure of the articulatory precision associated with each phonological class. The approach estimates 18 posterior probabilities from frames of 25 ms with a time-shift of 10 ms.

The previous representations define dynamic feature sequences, since the number of extracted frames depends on the duration of each speech recording. To obtain fixed-size representations, six statistical functionals were computed over time for every descriptor: mean, standard deviation, maximum, minimum, skewness, and kurtosis. This procedure generated response-level feature vectors, projecting the prosodic representation into $R^{78}$, the phonation representation into $R^{42}$, and the articulation representation into $R^{348}$. Participant-level representations were subsequently obtained by applying the same statistical aggregation strategy across all responses belonging to the same speaker.

#### 2.3.2 Time-frequency features

To capture temporal and spectral variations in the speech signal, the continuous wavelet transform was applied using the Mexican Hat wavelet, which is suitable for detecting localized variations in signal energy [13]. Speech signals were segmented into 64 ms frames, and wavelet coefficients were computed across multiple scales. For each frame and scale, energy-based representations were obtained from the squared magnitude of the wavelet coefficients. The resulting values were aggregated into a multi-scale representation of speech dynamics in the time-frequency domain. To obtain a fixed-size representation of the speech recording we follow the same approach based on statistical functionals presented in the Section 2.3.1.

#### 2.3.3 Deep learning-based speech embeddings

Given the well-known robustness of Wav2Vec 2.0 to represent speech signals, and also considering its multilingual extension especially fine-tuned for emotion recognition, namely XLSR-53, we decided to use it to evaluate its capability to model abnormal patterns related to distress in speech. Speech frames of 25 ms with a time-shift of 20 ms were transformed into 1024-dimensional embedding representations derived from the XLSR-53 architecture. These embeddings capture complex, data-driven acoustic and paralinguistic patterns directly from the raw audio waveform, providing an alternative to traditional handcrafted feature extraction methodologies. To obtain a fixed-size representation for each speech recording, we computed a temporal average over all frame-level embeddings, resulting in a single vector representation in $R^{1024}$ for each recording.

Audio recordings were processed through the frozen Wav2Vec 2.0 feature extractor. Embeddings were directly extracted from the last hidden state of the transformer. To obtain a single fixed-size vector per response, mean pooling was applied across all frames. Before classification, these embeddings were normalized using z-score standardization strictly within the inner cross-validation folds to prevent data leakage. Explicit dimensionality reduction was not applied to avoid discarding non-linear relationships. Instead, we selected classifiers natively suited for high-dimensional spaces: a Random Forest, which handles high dimensionality via feature subsampling, and a Deep Neural Network (DNN) designed to compress the feature space through bottleneck layers.

### 2.4 Classification

Machine learning models were used to evaluate the extracted features for the detection of psychological distress, considering both response-level and participant-level approaches.

The classification framework was selected according to the characteristics of each data representation and the corresponding sample size. Response-level classification involved thousands of speech responses, making Fully Connected Neural Networks (FCNNs) suitable for learning from a relatively large number of training instances. In contrast, participant-level handcrafted representations were derived from a substantially smaller number of participants, for which Support Vector Machines (SVMs) are well suited due to their robustness in high-dimensional, small-sample settings. Similarly, the embedding-based representations were evaluated using classification models appropriate for high-dimensional embedding vectors, following the corresponding experimental framework. Therefore, classifier selection was driven by methodological considerations rather than by the feature representation itself.

#### 2.4.1 FCNN (response-level)

At the response level, a fully connected neural network (FCNN) was implemented using a multilayer perceptron. This selection is motivated by the larger number of available samples, which allows the model to capture non-linear relationships in individual responses.

Hyperparameter optimization was performed using GridSearchCV. The search space included:

- Hidden layer configurations: (64), (16), (64,16)
- Activation functions: ReLU, tanh
- L2 regularization term (α): $10^{-5}$, $10^{-4}$, $10^{-3}$, $10^{-2}$
- Learning rate schedules: constant, invscaling, adaptive

Optimization was performed using the Adam solver with balanced accuracy as the selection metric.

#### 2.4.2 SVM (participant-level)

At the participant level, a support vector machine (SVM) classifier was employed.

Feature standardization was applied using z-score normalization. Hyperparameter optimization was performed using a two-stage procedure within a nested cross-validation framework. In the first stage, GridSearchCV was used for coarse exploration of the parameter space. Two kernel configurations were evaluated:

- Linear kernel: $C\in [10^{-6},10^{6}]$
- RBF kernel: $C\in [10^{-6},10^{6}]$, $\gamma \in [10^{-6},10^{6}]$

In the second stage, RandomizedSearchCV was applied to refine the search around the best parameter regions identified in the first stage, using exponential sampling. Class imbalance was handled using class_weight = balanced.

#### 2.4.3 Embedding-based models (XLSR-53)

For the classification of the XLSR-53 embeddings at the participant level, a separate classification framework was implemented. Two primary strategies were evaluated: a Random Forest classifier and a custom Deep Neural Network (DNN) implemented in PyTorch.

The Random Forest search space included the number of estimators (50, 100, 200, 300) and maximum depth (5, 10, 20, 25, None).

The DNN architecture consisted of batch normalization, a fully connected hidden layer, ReLU activation, and dropout, evaluating hidden dimensions (32, 64, 128), dropout rates (0.4, 0.6, 0.8), learning rates, and weight decay terms.

To ensure reproducibility, a fixed random seed (42) was applied across all data splits and model initializations. The custom DNN was optimized using Adam (learning rates $\in \{10^{-3},5\times 10^{-4}\}$, weight decay $\in \{10^{-3},10^{-2}\}$), with batch sizes of 32. Training was limited to a maximum of 200 epochs, incorporating early stopping with a patience of 15 epochs. All feature extraction and model training were executed on an NVIDIA Quadro RTX 5,000 GPU. Computationally, extracting XLSR-53 embeddings is highly resource-intensive due to the model size (>300 M parameters), whereas downstream classifier training (SVM, RF, DNN) is lightweight, requiring only minutes per fold.

### 2.5 Evaluation

The performance of the models was evaluated using a nested cross-validation design at the participant level. An outer 10-fold cross-validation scheme was used to estimate generalization performance on unseen participants. To prevent data leakage, the data splitting ensured that samples from the same participant did not appear simultaneously in both training and test sets.

Within each outer training fold, an inner 5-fold cross-validation split was used for hyperparameter optimization. This inner validation process ensured that model selection was performed without access to the outer test data.

For the response-level FCNN model, hyperparameters were tuned using GridSearchCV within the inner 5-fold cross-validation of each outer training fold.

For the participant-level SVM model, performance was estimated using the same nested cross-validation framework described above. Hyperparameter optimization was performed exclusively within the inner training folds, and final evaluation was carried out on the outer test folds.

For the deep-learned speech embeddings, a distinct nested cross-validation approach was implemented. Within each of the 10 outer folds, the training data was subjected to an 80/20 inner train-validation split exclusively for hyperparameter tuning. Furthermore, unlike the traditional models which were optimized for balanced accuracy, model selection for the deep embeddings was optimized directly based on the Area Under the ROC Curve (AUC) evaluated on this inner validation set, followed by final performance estimation on the isolated outer test fold. The complete nested cross-validation framework across feature representations is illustrated in Supplementary Figure S1.

Model performance was primarily evaluated using the F1-score, which was selected as the main comparative metric as it jointly accounts for both precision and recall, providing a more informative assessment of classification performance. Secondary evaluation metrics included accuracy, sensitivity, specificity, and the area under the ROC curve (AUC).

For each metric, the mean and standard deviation were reported.

## 3 RESULTS

This section presents the results obtained from all evaluated representations, including DisVoice, Phonet, and time-frequency features, considering both a response-level approach based on a fully connected network and a participant-level approach using an SVM classifier.

No feature-level fusion strategies (e.g., early or late fusion) were applied between representations, as preliminary experiments showed degraded performance when combining feature sets. Therefore, each speech representation was evaluated independently to preserve interpretability and avoid introducing additional dimensionality-related noise.

### 3.1 Response-level approach

The results at the response level are summarized in Table 2.

**Table 2:** Response-level results using a fully connected model.

Representation	F1-score	Accuracy	Sensitivity	Specificity	AUC
DisVoice—Prosody	0.47 ± 0.05	0.49 ± 0.04	0.49 ± 0.11	0.50 ± 0.07	0.47 ± 0.06
DisVoice—Phonation	0.46 ± 0.10	0.49 ± 0.07	0.47 ± 0.12	0.51 ± 0.09	0.49 ± 0.09
DisVoice—Articulation	0.39 ± 0.14	0.43 ± 0.09	0.40 ± 0.18	0.46 ± 0.14	0.41 ± 0.13
Phonet	0.43 ± 0.10	0.51 ± 0.07	0.46 ± 0.05	0.55 ± 0.10	0.43 ± 0.08
Time-Frequency	0.43 ± 0.08	0.47 ± 0.06	0.45 ± 0.10	0.50 ± 0.09	0.48 ± 0.07

At the response level, a comparative analysis of the different speech representations was conducted using the F1-score as the primary evaluation metric, as it provides a balanced measure between false positives and false negatives, enabling a more robust assessment of model performance. Overall, all methods exhibit limited performance, with F1-scores close to 0.5, indicating low discriminative capacity under this approach.This behavior is consistent across all evaluated representations, including DisVoice (prosody, phonation, and articulation), Phonet, and time-frequency-based features. Only minor differences are observed across representations. Prosodic features achieved the highest means F1-score, followed by phonation, while phonetic (Phonet) and time-frequency features showed slightly lower values. Articulation consistently presented the lowest results among the evaluated representations.

Performance variability across folds suggests sensitivity to local fluctuations in speech at the response level. These findings suggest that response-level representations have limited capacity to capture stable patterns associated with psychological distress.

### 3.2 Participant-level approach

The results at the participant level are summarized in Table 3.

**Table 3:** Participant-level results.

Representation	F1-score	Accuracy	Sensitivity	Specificity	AUC
DisVoice—Prosody	0.67±0.07	0.64±0.10	0.72±0.13	0.56±0.25	0.65±0.12
DisVoice—Phonation	0.59±0.16	0.61±0.14	0.57±0.18	0.66±0.20	0.65±0.16
DisVoice—Articulation	0.59±0.10	0.52±0.07	0.72±0.23	0.32±0.28	0.52±0.13
Phonet	0.54±0.20	0.53±0.17	0.59±0.26	0.46±0.17	0.54±0.16
Time-Frequency	0.48±0.16	0.52±0.13	0.47±0.18	0.56±0.21	0.58±0.14
XLSR-53 Embeddings (RF)	0.48±0.19	0.55±0.13	0.47±0.21	0.63±0.19	0.52±0.15
XLSR-53 Embeddings (DNN)	0.44±0.20	0.48±0.17	0.48±0.25	0.49±0.17	0.51±0.16

At a participant level, a consistent improvement in performance is observed across all speech representations compared to the response-level approach. This is likely explained due to the more information included when all responses are jointly analyzed, i.e., participant-level evaluation. In particular, prosodic features achieved the highest mean F1-score among the evaluated representations. Phonation and articulation features showed intermediate performance, while phonetic features (Phonet) and time-frequency representations exhibited lower performance.

Regarding the high-dimensional embeddings extracted via the XLSR-53 architecture, they were evaluated exclusively at the participant level utilizing the temporal aggregation strategy (mean matrix). Both Random Forest and a custom Deep Neural Network (DNN) were evaluated to handle the 1,024-dimensional feature space, aiming to contrast a robust tree-based ensemble approach against a highly non-linear deep architecture.

The Random Forest model achieved the most competitive performance within this framework, providing a better balance between sensitivity and specificity compared to the DNN. However, overall performance metrics remained close to chance, indicating challenges in adapting emotion-tuned embeddings to psychological distress detection without task-specific fine-tuning. Interestingly, the XLSR-53 embeddings evaluated at this level did not outperform handcrafted acoustic features under the evaluated setup. Despite establishing a robust modern baseline, the aggregated embeddings yielded an AUC of 0.52 and an F1-score of 0.48, placing their overall performance below that of prosodic and phonation dimensions.

Additionally, greater variability is observed in some representations, particularly in phonetic, time-frequency, and deep embeddings features, suggesting less stable behaviour and possibly a need for more data. Prosody achieved the highest mean F1-score and showed lower dispersion across folds, indicating greater consistency of the estimated performance. However, the difference between prosodic and phonation features was not statistically significant according to the Wilcoxon test. These findings suggest that participant-level analysis enables the capture of more robust speech patterns, reducing the impact of response-level variability and improving the overall discriminative capability of the models.

To assess whether the observed differences were statistically supported, Wilcoxon signed-rank tests were performed using the paired F1-scores obtained from the ten outer folds of the nested cross-validation procedure. In addition, 95% confidence intervals (CI) for the mean F1-score were computed from the same outer-fold results using the Student’s t-distribution. At the participant level, prosodic features achieved the highest mean F1-score among the handcrafted speech representations (0.667), followed by phonation features (0.595). However, the difference between both representations was not statistically significant (Wilcoxon signed-rank test, p=0.1875). The corresponding 95% confidence intervals were [0.615, 0.718] for prosody and [0.475, 0.716] for phonation. A second analysis was conducted to evaluate the impact of the aggregation strategy by comparing prosodic modeling at the participant and response levels. Participant-level prosodic modeling significantly outperformed response-level modeling (mean F1-score: 0.667 vs. 0.467; Wilcoxon signed-rank test, p=0.001953). The corresponding 95% confidence intervals were [0.615, 0.718] and [0.428, 0.507], respectively. Finally, participant-level prosodic features significantly outperformed the best-performing XLSR-53 embedding configuration (Random Forest classifier) (mean F1-score: 0.667 vs. 0.476; Wilcoxon signed-rank test, p=0.009766). The corresponding 95% confidence intervals were [0.615, 0.718] for prosodic features and [0.343, 0.610] for the XLSR-53 embeddings.

**Figure 1:**
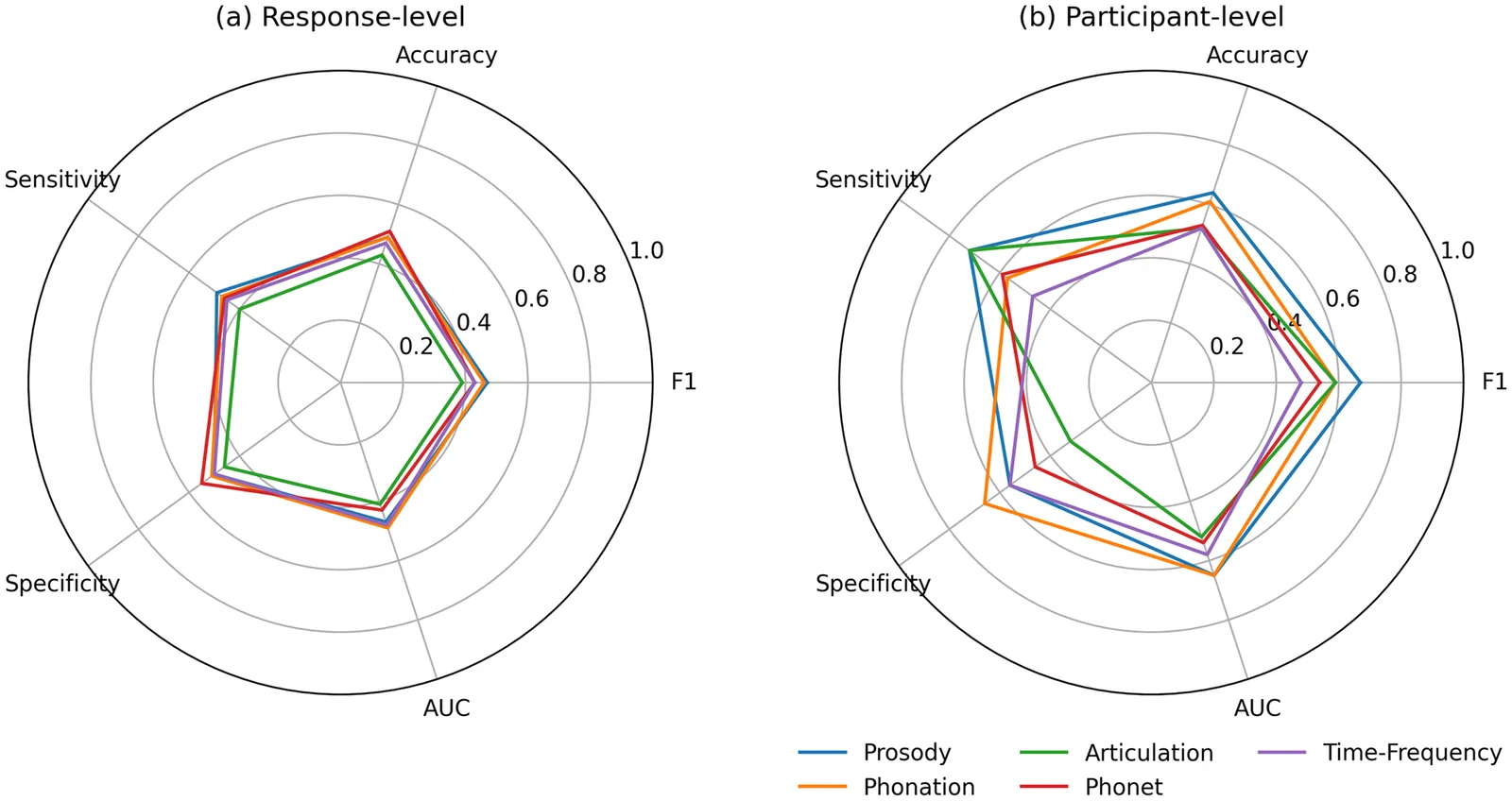
Comparison of speech representations at response-level **(a)** and participant-level **(b)**. Subfigure **(a)** shows the performance obtained using a fully connected neural network at the response level, while subfigure **(b)** shows the performance at the participant level using an SVM classifier (and Random Forest for deep embeddings). The radar plots summarize the behavior of all evaluated representations across F1-score, accuracy, sensitivity, specificity, and AUC.

Figure 1 presents a comparative visualization of all evaluated speech representations. Subfigure (a) illustrates the response-level results obtained using a fully connected neural network, while subfigure (b) shows the participant-level results based on an SVM classifier (and Random Forest for deep embeddings). This joint visualization facilitates a direct comparison between both analytical levels, making evident the differences in performance patterns across representations and evaluation metrics.

## 4 Discussion

The results obtained in the DAIC-WOZ corpus show that speech dimensions exhibit different behaviors in the detection of patterns associated with psychological distress. At the participant level, prosodic features achieved the highest mean F1-score among the evaluated speech representations. However, the difference between prosodic and phonation features was not statistically significant according to the Wilcoxon signed-rank test, indicating comparable performance between both speech dimensions within the evaluated dataset. These findings are consistent with previous studies showing that prosodic characteristics, such as intonation, rhythm, and intensity, as well as phonation-related aspects of voice production, are associated with emotional and affective states [3, 5].

From an interpretability perspective, these results suggest that the most discriminative speech dimensions may reflect relevant variations in speech production. However, these features should be interpreted as acoustic correlates of psychological distress rather than direct clinical biomarkers. Importantly, the objective of this study is comparative and methodological rather than clinical, and the reported performance should not be interpreted as diagnostic capability.

Furthermore, the better performance at the participant level suggests that this level of representation provides a more stable characterization of the speaker’s state. By aggregating multiple responses, the high intra-speaker variability present in isolated utterances is mitigated, supporting the usefulness of this participant-level analysis for a more reliable emotional state characterization. This result should be interpreted in the context of the experimental setup and comparative nature of the study.

Although the present study does not include explicit feature importance methods such as SHAP or permutation-based analysis, the comparison across predefined feature groups (prosody, phonation, articulation, phonetic, time-frequency, and deep embeddings) provides a structured view of the relative contribution of different speech representations.

From a clinical perspective, these findings are relevant in the context of the high comorbidity between post-traumatic stress disorder (PTSD) and major depressive disorder, with reported rates exceeding 50%, indicating that a substantial proportion of individuals with PTSD also present depressive symptomatology, and that both conditions frequently co-occur over time [14]. This overlap supports the use of a unified distress-related condition as the target class in this study, as it reflects a more realistic representation of shared affective and stress-related manifestations observed in clinical populations. While this strategy improves the feasibility of the analysis and enhances the detection of general speech-based patterns associated with psychological distress, it also reduces diagnostic specificity across individual disorders. In this sense, speech alterations may be better interpreted as indicators of a broader distress-related spectrum rather than as markers of strictly isolated diagnostic categories. Furthermore, although PTSD and depression remain clinically distinct disorders, they may share partially overlapping speech manifestations, particularly in prosodic and phonation-related dimensions. The participant-level approach may therefore be more aligned with real-world applications, where the objective is to characterize an individual’s overall speech profile rather than isolated responses. However, any potential clinical translation of these findings requires further external validation, calibration across datasets, and prospective evaluation before being considered for deployment in clinical settings.

Prior studies using the DAIC-WOZ corpus have reported a wide range of results depending on the speech representation and modeling strategy. Classical work such as Gratch et al. [8] demonstrated that voice-quality characteristics can distinguish participants with depression and PTSD from healthy controls separately, highlighting the relevance of low-level acoustic cues. More recent representative studies, such as Al Hanai et al. [10], reported an F1-score of approximately 0.67 for audio-only models and 0.77 when combining speech and text in a multimodal architecture. Similarly, Dumpala et al. [15] obtained balanced accuracy values around 66% using speaker embeddings combined with acoustic features. AAlthough previous studies reported different evaluation metrics and experimental settings, the participant-level performance achieved by the prosodic features in this study is broadly consistent with previously reported speech-based results on the DAIC-WOZ dataset. However, direct comparisons should be interpreted with caution, as most prior studies focus exclusively on depression detection and typically evaluate a limited number of representations or modeling strategies.

Importantly, the results obtained in this study also indicate that combining multiple feature sets did not necessarily lead to improved performance. Although different representations, including DisVoice speech dimensions, Phonet, and time-frequency features, capture complementary aspects of speech, their integration did not consistently translate into higher discriminative performance. This suggests that increased feature complexity does not necessarily improve classification outcomes in the absence of task-specific optimization or appropriate feature selection strategies. Overall, the reported performance differences should be interpreted as methodological observations within the DAIC-WOZ dataset rather than as evidence of superiority between approaches.

The observation that deep learning-based embeddings specifically tuned for speech emotion recognition did not surpass traditional handcrafted features at the participant level within the DAIC-WOZ corpus is an important finding. This suggests that while pre-trained transformer-based architectures excel at capturing general acoustic and emotional patterns, they may require explicit, task-specific fine-tuning to effectively isolate the subtle vocal biomarkers associated with psychological distress. Handcrafted features such as prosody and phonation achieved higher mean performance than the evaluated XLSR-53 embeddings in this specific clinical context. These findings suggest that for specialized medical applications, domain knowledge encoded in acoustic parameters can still offer competitive advantages over generalized deep learning representations.

The limited performance of deep-learned embeddings stems from two methodological constraints: the lack of fine-tuning and the temporal pooling strategy. First, fine-tuning a massive Transformer architecture on a limited dataset of 125 participants poses a severe risk of overfitting. Consequently, the model was used strictly as a frozen feature extractor. Without task-specific fine-tuning, the embeddings may have primarily captured generalized emotional variations rather than targeted clinical pathology. Second, applying mean pooling across all frames likely diluted localized distress markers. Psychological distress often manifests in sporadic, short-duration events like micro-pauses or transient vocal tremors. Averaging the last hidden state across the entire utterance reduces the signal-to-noise ratio of these critical anomalies. As a result, stable handcrafted features outperformed the deep representations.

### 4.1 Limitations

This study presents several limitations that should be acknowledged. First, the analysis is based on a single corpus (DAIC-WOZ), with internal validation only and no external dataset evaluation, which limits generalizability across populations and recording conditions. The dataset also includes variability in interview conditions, acoustic noise, and recording quality, as the recordings were not collected under a controlled protocol.

Although the dataset contains 3,069 speech responses, the participant-level classification task is ultimately based on 125 individuals. Consequently, the effective sample size remains relatively limited, which may reduce statistical power, increase uncertainty in the estimated performance metrics, and contribute to the variability observed across cross-validation folds. This limitation may contribute to the relatively wide confidence intervals and the variability observed for some representations.

In addition, the classification task was formulated as a broad psychological distress condition by grouping participants with depressive symptoms, PTSD symptoms, or both into a single category. Although this formulation is consistent with the study objective and facilitated a more balanced classification task, it introduces label heterogeneity due to the clinical differences between depression and post-traumatic stress disorder. These conditions exhibit distinct symptoms and different speech manifestations, which increases within-class variability and may reduce class separability. This factor could have negatively influenced classification performance.

Furthermore, only one randomly sampled subset of healthy controls was used throughout the experiments. Although the sampling procedure was designed to obtain a balanced participant-level dataset, the use of a single subset may introduce residual selection bias. Future studies should evaluate the robustness of the findings across multiple balanced subsets to further assess the potential impact of participant selection on classification performance.

The study does not include calibration analysis or decision-curve evaluation, limiting insights into clinical decision thresholds. Furthermore, longitudinal data are not available, preventing temporal modeling of symptom progression.

Finally, because a nested cross-validation framework was employed, hyperparameter selection was dynamic across training folds. Therefore, single optimal hyperparameter values are not reported, although the complete search spaces and random seeds have been detailed to ensure methodological reproducibility. In addition, the study was intentionally restricted to speech-based representations in order to provide a controlled comparison across multiple speech feature sets. Consequently, multimodal information such as text, facial behavior, or physiological signals was not incorporated. Although this design facilitates the interpretation of the relative contribution of speech representations, previous studies on the DAIC-WOZ corpus have shown that multimodal approaches can achieve higher performance than speech-only systems.

### 4.2 Conclusion

Within the evaluated DAIC-WOZ corpus, participant-level prosodic features achieved the highest mean performance among the tested feature sets, while phonetic, time-frequency, and deep-learned embeddings did not outperform the best handcrafted acoustic baseline under the evaluated experimental setup. Within the evaluated experimental setting, participant-level aggregation consistently outperformed response-level modeling, indicating that the way speech is grouped and evaluated may be more influential than simply increasing representational complexity. The findings also suggest that transparent preprocessing strategies and leakage-aware evaluation protocols play a more decisive role than adopting increasingly complex feature families. Although the reported performance is not sufficient to support clinical decision making, the study provides a methodological contribution within the context of the DAIC-WOZ corpus by showing that robust modeling pipelines are essential for advancing speech-based analysis toward more reliable research frameworks for speech-based analysis.

## Data Availability

The DAIC-WOZ dataset is available for research purposes under controlled access. The data were obtained through an official access link provided by the dataset authors and are subject to their terms and conditions of use. Requests for access to the dataset should be directed to the University of Southern California Institute for Creative Technologies (https://dcapswoz.ict.usc.edu).

## Footnotes

^1^ https://dcapswoz.ict.usc.edu/wwwdaicwoz/
