# Supplementary material for: Automatic analysis of speech representations to assess psychological distress
Source: Front Digit Health. 2026 Aug 12;8:1886893. doi: 10.3389/fdgth.2026.1886893 (PMC13508159; doi:10.3389/fdgth.2026.1886893)
Supplement: Supplementary file 1 [file Datasheet1.pdf]

Supplementary Material

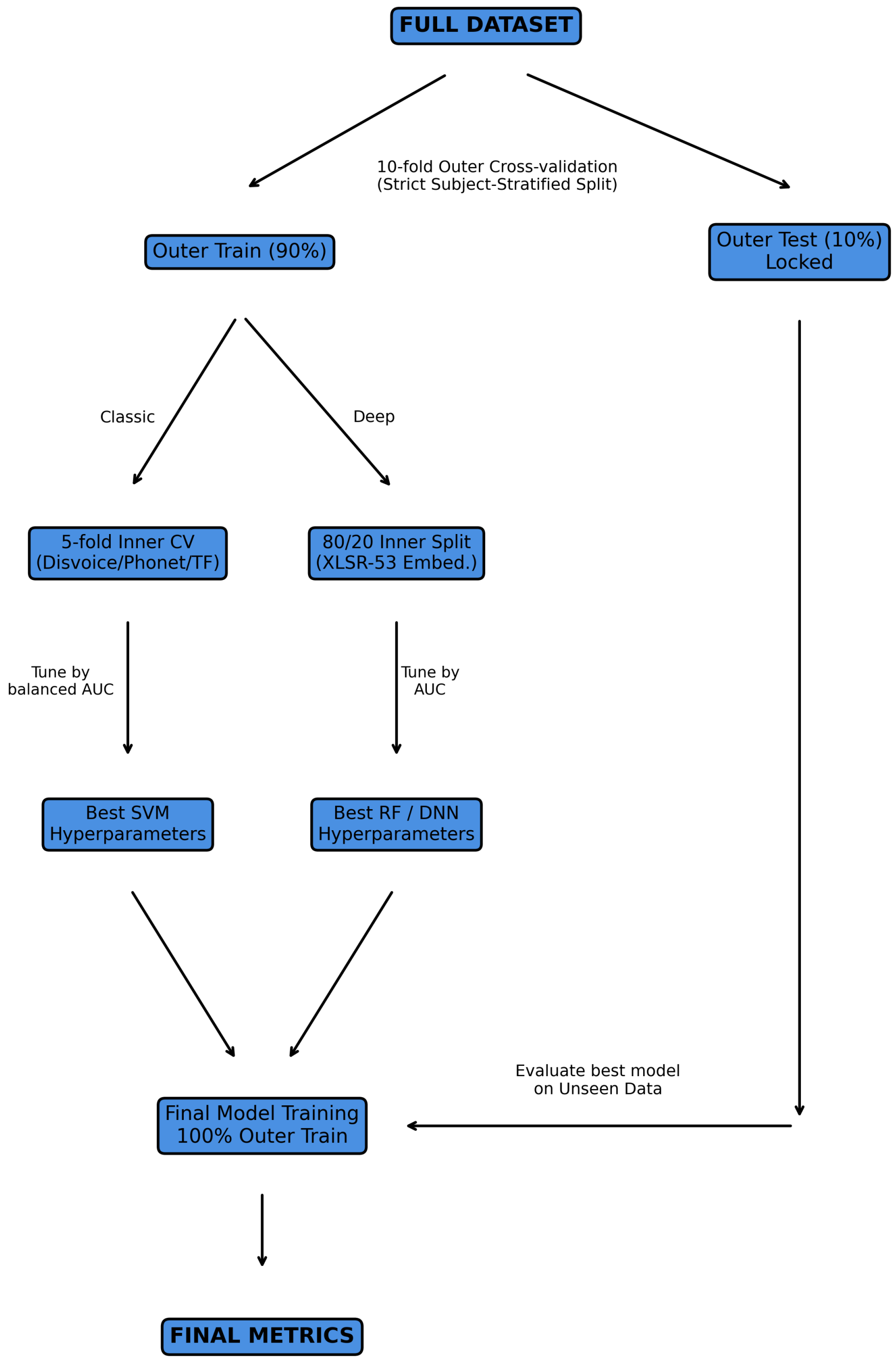

Figure S1. Overview of the nestedcross-validation strategy.The outerloop ensures strict subject-stratified splitting to isolate the test set.In the inner loop, traditional features utilize a 5-fold cross-validation for hyperparameter tuning, while deep-learned embeddings employan 80/20 train-validation split optimizing for AUC.
